# Morphometric Analysis of Connective Tissue Sheaths of Sural Nerve in Diabetic and Nondiabetic Patients

**DOI:** 10.1155/2014/870930

**Published:** 2014-07-24

**Authors:** Braca Kundalić, Slađana Ugrenović, Ivan Jovanović, Natalija Stefanović, Vladimir Petrović, Jasen Kundalić, Vesna Stojanović, Vladimir Živković, Vladimir Antić

**Affiliations:** ^1^Department of Anatomy, Faculty of Medicine, University of Niš, Boulevard Dr. Zoran Đinđić 81, 18000 Niš, Serbia; ^2^Chair of Medical Subjects, Faculty of Sport and Physical Education, University of Niš, Čarnojevićeva Street 10A, 18000 Niš, Serbia; ^3^Department of Histology and Embryology, Faculty of Medicine, University of Niš, Boulevard Dr. Zoran Đinđić 81, 18000 Niš, Serbia; ^4^Faculty of Medicine, University of Niš, Boulevard Dr. Zoran Đinđić 81, 18000 Niš, Serbia

## Abstract

One of the most common complications of diabetes mellitus is diabetic neuropathy. It may be provoked by metabolic and/or vascular factors, and depending on duration of disease, various layers of nerve may be affected. Our aim was to investigate influence of diabetes on the epineurial, perineurial, and endoneurial connective tissue sheaths. The study included 15 samples of sural nerve divided into three groups: diabetic group, peripheral vascular disease group, and control group. After morphological analysis, morphometric parameters were determined for each case using ImageJ software. Compared to the control group, the diabetic cases had significantly higher perineurial index (*P* < 0.05) and endoneurial connective tissue percentage (*P* < 0.01). The diabetic group showed significantly higher epineurial area (*P* < 0.01), as well as percentage of endoneurial connective tissue (*P* < 0.01), in relation to the peripheral vascular disease group. It is obvious that hyperglycemia and ischemia present in diabetes lead to substantial changes in connective tissue sheaths of nerve, particularly in peri- and endoneurium. Perineurial thickening and significant endoneurial fibrosis may impair the balance of endoneurial homeostasis and regenerative ability of the nerve fibers. Future investigations should focus on studying the components of extracellular matrix of connective tissue sheaths in diabetic nerves.

## 1. Introduction

Diabetic polyneuropathy is the most common neurological complications of diabetes mellitus. More than 80% of patients with diabetic neuropathy have developed distal symmetrical diabetic neuropathy (DSDN) [1], which is length dependent, due to early onset in the longest nerve fibers which are situated in the feet, then affecting more proximal parts of the lower limbs and distal parts of upper limbs eventually. This form of polyneuropathy affects at least 50% of diabetic patients and is the leading cause of foot amputation [2]. One of the major chronic diabetic complications involving diabetic neuropathy, retinopathy, and nephropathy is microangiopathy [3]. Abnormalities reported in diabetic neuropathy include axonal degeneration in nerve fibers, primary demyelination resulting from Schwann cell dysfunction, secondary segmental demyelination related to impairment of the axonal control of myelination, remyelination, proliferation of Schwann cells, atrophy of denervated bands of Schwann cells, onion-bulb formations, and hypertrophy of the basal lamina [4]. Morphological alterations of endoneurial microvessels have been described in chronic ischaemic/hypoxic neuropathy, for example, diabetic neuropathy [5–7]. In diabetic nerves, microvascular changes precede the development of neuropathy and are related to the severity of neuropathy, although their implications and roles in the development of neuropathy remain uncertain [8]. Peripheral neuropathy can also be caused by nondiabetic peripheral vascular disease in chronically ischaemic limbs [9].

Three connective tissue sheaths of peripheral nerves, epi-, peri-, and endoneurium are continuation of the dura mater, arachnoid mater, and pia mater, respectively. In addition to protecting the nerve fibers from compression and stretching, these layers of connective tissue also have a distinct role in providing optimal environment for the normal function and regeneration of nerve fibers. The epineurium is the outermost connective tissue sheath that surrounds peripheral nerve. The vasa nervosum enters the epineurium branching to the network of arterioles and venules which, within perineurium, penetrate into fascicles [10]. The perineurium is part of the connective tissue layers of the peripheral nerve which surrounds and protects nerve fascicles. It is a lamellated structure made up of concentric layers which are separated by interlamellar clefts rich with collagen fibrils [11]. Histochemical studies showed a variety of phosphorylating enzymes inside perineurial cells which have a high level of ATPase activity. Therefore the perineurial cells make no simple passive restrictive barrier but a metabolically active diffusion barrier [12]. Endoneurium is the innermost connective tissue sheath that covers and supports individual nerve fibres within the fascicle. Nerve fibers appear to be hermetically sealed between basement membrane of endoneurial capillaries and lamellar perineurial sheath, so that each change in neural layers may influence their function and process of regeneration. Therefore the aim of our study was to perform morphological and morphometric analysis of epi-, peri-, and endoneurial sheaths of sural nerve in diabetic patients.

## 2. Materials and Methods

The examination was carried out on 15 sural nerves routinely dissected at the Institute of Forensic Medicine, Niš, and after below-knee and above-knee amputations at the Vascular Surgery Clinic, Clinical Centre Niš. The samples of sural nerve were taken in each subject in the following way: 5 cm long skin cut was made between lateral malleolus and calcaneal tendon; after removal of outer skin layer and subcutaneous tissues 3 cm long part of sural nerve trunk was harvested and afterwards fixed in 10% neutral-buffered formalin for 24 hours within 1 hour after surgical removal. The specimens were embedded in paraffin using standard procedures. Serial transverse sections were 5 *μ*m thick and cut on a microtome with a disposable blade for hematoxylin-eosin and Masson's trichrome staining.

All samples are divided into 3 groups, 5 cases each. Mean age between groups was not significant. The first group consisted of patients with type II diabetes (mean age 70 ± 12 years), with a range of disease duration from 8 to 20 years. Effects of diabetic neuropathy were the reason for surgical lower limb amputation. The patients diagnosed as having peripheral vascular disease (PVD) were in the second group (mean age 75 ± 3 years). The amputations were performed due to the lower limb and foot ulcerations and necrosis, with a history of vascular reconstruction. Control group consisted of tissue samples obtained postmortem, within 12 h of death. All the patients (mean age 72 ± 8) had no type II diabetes or PVD in the medical history.

**Figure 1:**
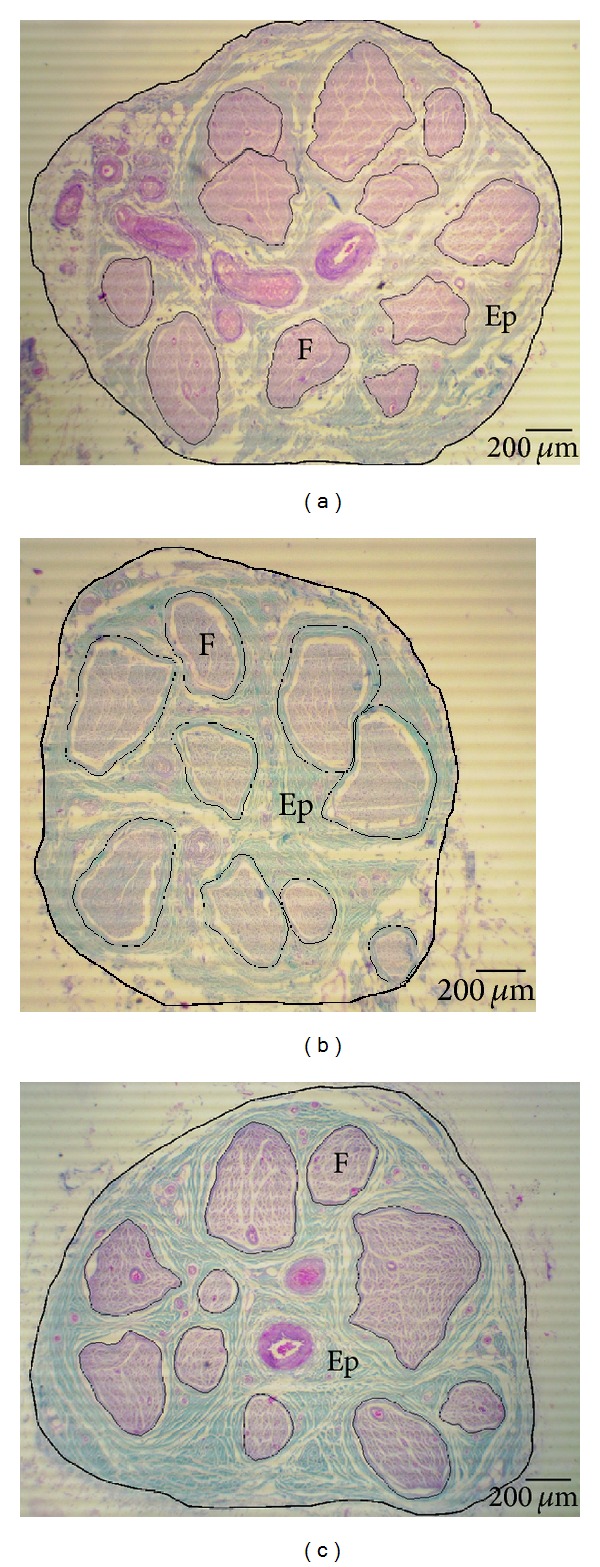
Cross section of sural nerve of diabetes group (a), PVD group (b), and control group (c). Outer contour of total nerve cross-sectional area and every fascicle area is rounded with black line. F: fascicle, Ep: epineurium; 40x lens magnification. Masson's trichrome staining.

**Figure 2:**
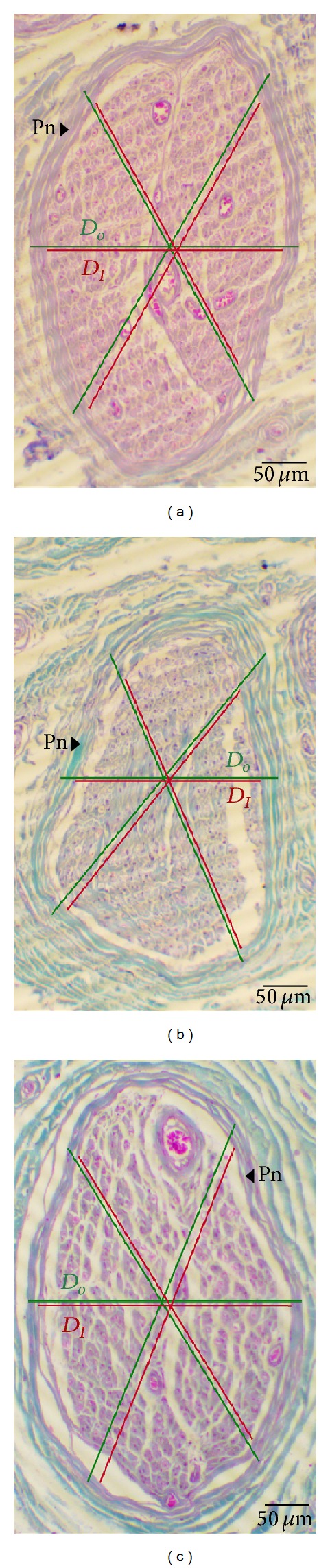
Cross section of sural nerve fascicle in diabetes group (a), PVD group (b), and control group (c). The image measurement of outer (*D*_*O*_) and inner diameter of the fascicle (*D*_*I*_) is marked with lines at three different points; Pn: perineurium; 100x lens magnification. Masson's trichrome staining.

**Figure 3:**
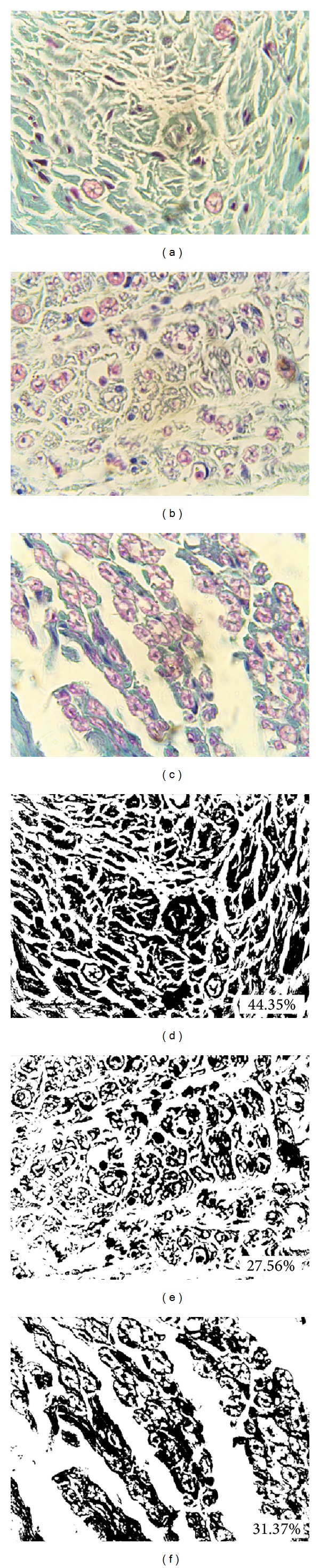
Quantification method for percentage of endoneurial connective tissue of sural nerve in diabetes group ((a) and (d)), PVD group ((b) and (e)), and control group ((c) and (f)); 1000x lens magnification. Masson's trichrome staining ((a), (b), and (c)).

Morphologic analysis of all sections is done by microscope “Olympus C011” after checking for artifacts and pathologic appearance under light microscopy. We investigated number and morphology of fasciculi, epineurial and perineurial sheath, and endoneurial content. Afterwards the images were made using ×40, ×100, and ×1000 objective on 5-megapixel colour digital microscopy camera. The images were used for morphometric analysis by ImageJ image analyzing software (http://rsb.info.nih.gov/ij/). Prior to every measurement spatial calibration is done using object micrometer (1 : 100) for every magnification. At the lowest magnification values of total nerve cross-sectional area are measured, as well as total fascicular and total epineurial area (Figure 1). Taking into consideration that there are literature data [13, 14] about the influence of fascicle's size on perineurial thickness, we also measured outer (distance between two spots on the opposite sides of outer perineurial surface which pass through the center of fascicule—*D*_*O*_) and inner (distance between opposite two spots on the inner perineurial surface which pass through the center of the fascicule—*D*_*I*_) diameter of the measured fascicles in order to calculate perineurial index (P index) according to the formula P index = [(*D*_*O*_ − *D*_*I*_)/*D*_*O*_] × 100 [15] (Figure 2). At the highest magnification we measured endoneurial connective tissue on 10 randomly chosen areas of great number of fasciculi for every examined case (Figures 3(a), 3(b), and 3(c)). Analysis is done using the option Image/Adjust/Color Threshold, sampling the colour at minimum three points. After converting the image to binary, we used the option Measure to calculate the area fraction on the examined areas. A total mean endoneurial connective tissue area was then calculated for each case (Figures 3(d), 3(e), and 3(f)).

## 3. Statistical Methods

To test the difference in mean values for statistical significance, we used Student's *t*-test for small independent sample, ANOVA, and Tukey's post hoc test. This was done using SPSS for Windows version 20.

## 4. Results

**Figure 4:**
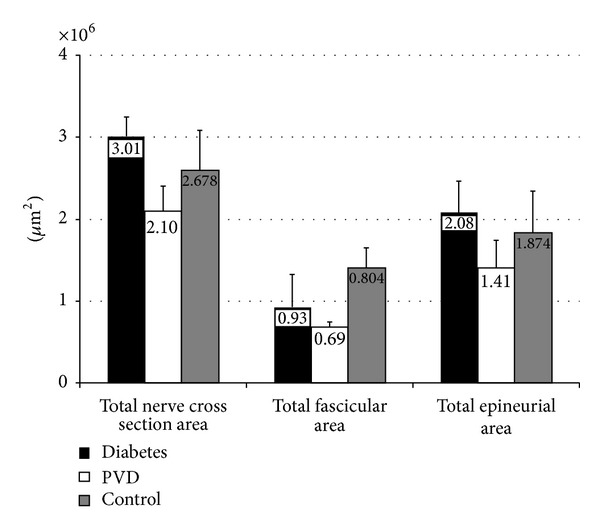
Graphic overview of mean value of total nerve cross-sectional area, fascicular area, and epineurial area of sural nerve in diabetes, PVD, and control group.

After morphometric analysis we observed that the highest mean value of total neural and epifascicular area was in the diabetic group (Table 1), which was significant in comparison to nondiabetic PVD group (*P* < 0.05 and *P* < 0.01, resp.). The control group showed higher values of all areas comparing to PVD group but no significance (Figure 4).

**Figure 5:**
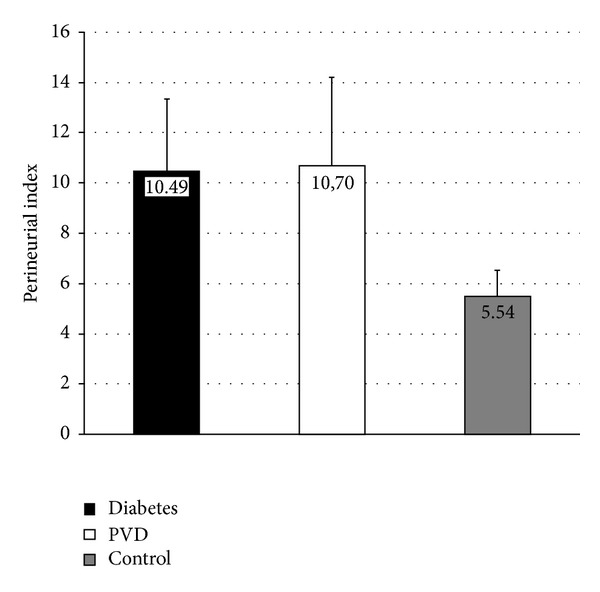
Graphic overview of mean value of perineurial index of sural nerve in diabetes, PVD, and control group.

After quantitative analysis using Tukey's post hoc test, the results showed that there were significantly higher values of perineurial index in both diabetic and PVD groups comparing to control group (*P* < 0.05 and *P* < 0.01, resp.) (Table 1). There was small difference between diabetic and nondiabetic group which showed no significance (Figure 5).

**Figure 6:**
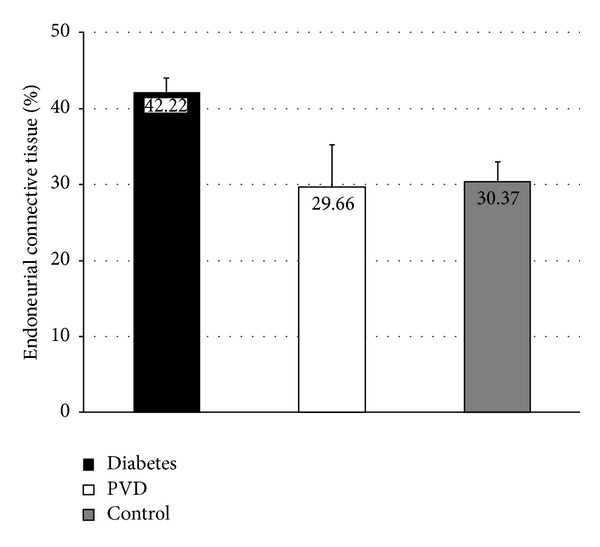
Graphic overview of mean value of endoneurial connective tissue of sural nerve in diabetes, PVD, and control group.

The relationship between percentages of endoneurial connective tissue was examined using Tukey's post hoc test. Our measurements showed that the highest percentage of connective tissue was present in diabetic group, and it was significantly higher than in PVD (*P* < 0.001) and control group (*P* < 0.01) (Table 1). Comparison between PVD group and control group showed no significance (Figure 6).

## 5. Discussion

Being the outermost layer of nerve, the epineurium is most prone to the macrovascular changes that happen in diabetes and peripheral vascular disease. Those changes are identified as occlusion of blood vessels and thrombosis and consequently impaired nerve blood flow, hypoperfusion, endothelial duplication, basement membrane thickening, and intima cells proliferation [15, 16]. Tesfaye et al. [17] investigated epineurial blood vessels in sural nerves of diabetic patients and found arteriolar attenuation with venous tortuosity and distension. They confirmed the presence of active epineurial arteriovenous shunting in diabetic subjects that may result in reduction of endoneurial blood flow. Llewelyn et al. [18] reported inflammatory reaction in epineurial vessels of cutaneous nerve of the thigh in diabetic neuropathy. Beside blood vessels, lymphatic vessels may also occur in higher number as a result of the increase of epineurial vascularization. Recent study of Agliano et al. [19] showed that epineurial lymphatics may also be dilated and replete with mononuclear cells as a result of chronic inflammation or ischemia. Our findings suggest that there is difference in epineurial compartment in diabetic nerves compared to those suffering vascular changes. Significant greater amount of connective tissue may be explained with metabolic disorders that are present in hyperglycemic state which consequently provoke inflammation which, being chronic, induces fibrotic response and excess production and deposition of proteins of extracellular matrix, while in PVD blood vessels they are occluded which leads to ischemia and cell atrophy.

Our analysis shows that there is significant higher perineurial index in both diseased groups compared to control one. Higher perineurial index indicates that thickening of perineurial ensheathment is present in diabetes and peripheral vascular disease cases. The study of El-Barrany et al. [20] showed several ultrastructural changes in the basement membrane of the perineurial cells of the diabetic nerves such as thickening. It also showed increased vacuoles and pinocytotic vesicles in the cellular layers of the perineurium. Consequently, these changes may suggest that permeability of the blood-nerve barrier was increased in the diabetic group. Hyperglycemia, which is the major metabolic abnormality of diabetes, has been shown to produce an upregulation of several major basement membrane components, including collagen IV and fibronectin [21, 22]. Hill and Williams [21, 23] detected with electron micrographs significantly thicker perineurial basement membrane of sural nerve in diabetic group compared to control one, but immunohistochemical analysis did not confirm that the thickening is caused by increased expression of collagen type IV, laminin, and fibronectin. In continuation of previous studies Hill [24] analyzed the presence of laminin, tenascin, and collagen types IV, V, and VI both in perineurium and in endoneurium of diabetic and nondiabetic cases. In this study the aforesaid author detected only significantly higher presence of collagen type VI in the perineurium of diabetic cases. It is apparent that finding the cause of thickening of the perineurial layer in diabetic cases needs further investigations.

Hyperglycemia is responsible for inducing oxidative stress and the polyol pathway. Higher levels of sorbitol and, consequently, fructose produced through polyol pathway are associated with impaired regulation of protein kinase C and Na^+^/K^+^-ATPase which leads to nerve dysfunction [25]. Excess glucose reacts with proteins forming advanced glycation end products (AGEs). Prime targets are proteins of extracellular matrix, particularly basement membrane proteins, such as types I, III, IV, and VI collagen, fibronectin, and laminin [26, 27]. Accumulation rate of AGEs is accelerated by hyperglycemia and they are shown in various organs, such as kidney, retina, heart, or atherosclerotic plaques [26, 28]. Clinical and experimental studies showed increased endoneurial collagen, reduplication of basement membranes around endoneurial capillaries, and thickening of basal lamina [29, 30]. Tubulin and neurofilament in the axons and myelin protein in Schwann cells may be modified by AGEs in nerve fibers, while collagen, laminin, and fibronectin were shown to be glycated in basement membrane and extracellular matrix [31]. Glycated laminin and fibronectin have been shown to be responsible for failure of axonal regeneration in rat diabetic sciatic nerves and decreased ability of rat sensory neurons to extend axons [32]. Regeneration of axons may be noticed alongside degenerating neurites in uninjured diabetic nerves, but with disease progressing a number of regenerating axons declines [33], which was also observed after sural nerve biopsy [34]. Recent findings showed no significant change in levels of laminin and collagen IV in the endoneurium of diabetic nerves, but upregulation of tenascin and types V and VI collagen in response to hyperglycemia was confirmed [24]. Excess collagen accumulation in endoneurial compartment is harmful for nerve fibre regeneration, as collagen fibrils are deposited inside Schwann cell basal laminal tubes, which are responsible for nerve fiber elongation, and therefore may prevent axon growth [35]. In our study endoneurial fibrosis is significantly higher only in diabetic group, which is in accordance with the results and conclusions of the investigations mentioned previously.

After morphometric analysis we can conclude that diabetes cases have significant thickening of perineurial sheath, along with endoneurial fibrosis, in comparison to control group. However epineurial ensheathment is significantly thicker only in diabetes cases comparing to PVD cases. Obtained results point to remarkable remodelling of connective tissue sheaths in diabetic neuropathy which will lead our further studies to the analysis of the components of connective tissue sheaths in diabetic nerves, such as various types of collagen and also chondroitin sulfate.

## Figures and Tables

**Table 1:** Results of morphometric analysis of sural nerve in diabetes, PVD, and control group.

Group	Diabetic patients	PVD patients	Control
Number of cases	5	5	5
Mean age	70 ± 12	75 ± 3	72 ± 8
Mean number of fasciculi	10.6 ± 4.7	7.4 ± 1.7	8.6 ± 2.7
Total nerve cross section area (*μ*m^2^)	3.01 × 10^6^ ± 0.24 × 10^6^**	2.1 × 10^6^ ± 0.3 × 10^6^	2.678 × 10^6^ ± 0.57 × 10^6^
Total fascicular area (*μ*m^2^)	0.93 × 10^6^ ± 0.4 × 10^6^	0.69 × 10^6^ ± 0.06 × 10^6^	0.804 × 10^6^ ± 0.19 × 10^6^
Total epineurial area (*μ*m^2^)	2.08 × 10^6^ ± 0.39 × 10^6^*	1.41 × 10^6^ ± 0.33 × 10^6^	1.874 × 10^6^ ± 0.55 × 10^6^
P index	10.49 ± 2.86^†^	10.70 ± 3.52^†^	5.54 ± 1.05
Percentage of endoneurial connective tissue	42.22 ± 1.76^∗∗∗,††^	29.66 ± 5.57	30.37 ± 2.67

*P < 0.05 versus PVD.

**P < 0.01 versus PVD.

***P < 0.01 versus PVD.

^†^ *P* < 0.05 versus control.

^††^ *P* < 0.01 versus control.
